# Genetic Structure of Pelagic and Littoral Cichlid Fishes from Lake Victoria

**DOI:** 10.1371/journal.pone.0074088

**Published:** 2013-09-06

**Authors:** Miyuki Takeda, Junko Kusumi, Shinji Mizoiri, Mitsuto Aibara, Semvua Isa Mzighani, Tetsu Sato, Yohey Terai, Norihiro Okada, Hidenori Tachida

**Affiliations:** 1 Department of Biology, Faculty of Sciences, Kyushu University, Fukuoka, Japan; 2 Graduate School of Bioscience and Biotechnology, Tokyo Institute of Technology, Yokohama, Japan; 3 Tanzania Fisheries Research Institute (TAFIRI), Mwanza, Tanzania; 4 Department of Life Sciences, National Cheng Kung University, Tainan, Taiwan; University of Konstanz, Germany

## Abstract

The approximately 700 species of cichlids found in Lake Victoria in East Africa are thought to have evolved over a short period of time, and they represent one of the largest known examples of adaptive radiation. To understand the processes that are driving this spectacular radiation, we must determine the present genetic structure of these species and elucidate how this structure relates to the ecological conditions that caused their adaptation. We analyzed the genetic structure of two pelagic and seven littoral species sampled from the southeast area of Lake Victoria using sequences from the mtDNA control region and 12 microsatellite loci as markers. Using a Bayesian model-based clustering method to analyze the microsatellite data, we separated these nine species into four groups: one group composed of pelagic species and another three groups composed mainly of rocky-shore species. Furthermore, we found significant levels of genetic variation between species within each group at both marker loci using analysis of molecular variance (AMOVA), although the nine species often shared mtDNA haplotypes. We also found significant levels of genetic variation between populations within species. These results suggest that initial groupings, some of which appear to have been related to habitat differences, as well as divergence between species within groups took place among the cichlid species of Lake Victoria.

## Introduction

A large number of cichlid fish species have been identified in each of the three great lakes of East Africa, Lakes Tanganyika, Malawi, and Victoria. Turner et al. [1] have estimated that there are approximately 250, 700 and 700 cichlid species endemic to Lakes Tanganyika, Malawi, and Victoria, respectively. Moreover, these species possess a wide variety of adaptations to specific environments, which seem to have developed over fairly short periods of time [2]. These adaptations have often involved changes to morphology and sensory organ structure, and they appear to have occurred independently within each lake [3]. In Lake Victoria, for example, the species flock of endemic cichlids is thought to be either monophyletic [4] or of hybrid origin from colonizing lineages [5] and includes morphologically and ecologically diverse species [6]. Furthermore, clear examples of ecological speciation are known to have occurred for the cichlids in this lake (e.g., [7], [8]). Therefore, these species provide us with an excellent opportunity to study adaptive radiation.

An important question with respect to adaptive radiation is how its initial stages are affected by different habitats. Using ultrametric trees of the Lake Victoria radiation, Seehausen et al. [5] inferred that the radiation can be thought of as a starburst pattern with either very short or no branches separating any two speciation events. In this case, species groupings based on habitat would not be apparent. On the other hand, Danley et al. [9] proposed that adaptive radiation first occurred by adaptation to different habitats – rocky and sandy habitats, in the case of Lake Malawi – followed by diversification with respect to trophic morphology and male nuptial color within each habitat. In this scenario, species groupings based on habitat would be apparent even at early stages and could be identified as a hierarchical genetic structure related to habitat. Danley et al. [9] proposed this scenario of evolutionary radiation based on the phylogenetic relationships between cichlid species in Lake Malawi, and they also cited examples from other species groups to suggest that this may represent a general mode of adaptive diversification. However, because many species may have gone extinct following the initial burst of adaptive radiation [2], it would be difficult to reconstruct the initial stages of diversification by examining only surviving species generated by older adaptive radiations.

The species flock of cichlids found in Lake Victoria is an excellent group of organisms with which to investigate the initial stage of adaptive radiation. As mentioned above, a variety of species adapted to different habitats exist [10], and the species flock is thought to have diverged over the last 100,000 years or less [4], [11]. Therefore, it may still be possible to infer the initial stage of adaptive radiation. However, the fact that the diversification of the species flock within Lake Victoria occurred so recently also poses a problem. Because the speciation events were so recent, genetic differentiation between species can be weak, and it is difficult to infer phylogenetic relationships within the species flock using neutral markers [5], [12], [13]. Indeed, Samonte et al. [14] have suggested that gene flow between species can be as extensive as flow between local populations of the individual species within Lake Victoria. However, our previous population genetic studies based on many individuals of pelagic cichlid species from Lake Victoria found significant, albeit weak, genetic differentiation between these species [15], [16]. Therefore, we propose that, by sampling many individuals from several species living in different habitats, we may be able to infer diversification patterns from the initial stage of adaptive radiation using neutral markers. Alternatively, this can be achieved using many more markers with a smaller number of samples for each species. Indeed, Bezault et al. [17], using amplified fragment length polymorphism (AFLP) markers, and Wagner et al. [18] and Keller et al. [19], using restriction-site-associated DNA (RAD) markers, have recently found significant differentiation between the cichlid fish species of Lake Victoria.

In this study, we genotyped populations of seven littoral cichlid species collected from the southern part of Lake Victoria using a mitochondrial marker and 12 microsatellite markers that were developed by Maeda et al. [20]. Furthermore, we combined these data with genetic information gathered previously from two pelagic species using the same set of markers [15]. By analyzing this dataset from nine species living in different habitats, we were able to address the following questions. (1) Does a hierarchical genetic structure for the cichlid fishes of Lake Victoria exist? (2) If so, is this hierarchical structure related to habitat? (3) Finally, are there further genetic substructures within these cichlid fishes, and how might these relate to species?

## Materials and Methods

### Ethics Statement

This study was conducted in collaboration with the Tanzania Fisheries Research Institute (TAFIRI), which also provided us with logistical support, including permissions for the field studies. In the field studies, we complied with local legislation and the Convention on Biological Diversity and the Convention on the Trade in Endangered Species of Wild Fauna and Flora. The animal protocols and procedures were approved by the Institutional Animal Care and Use Committee of Tokyo Institute of Technology.

### Sampling and DNA Extraction

We collected specimens of seven littoral cichlid species from the southern part of Lake Victoria between September 2004 and November 2006. All fish were collected by M. A., T. S. and S. M. All specimens were collected by gill net (1.5-m height) or angling from a depth of 0–10 m. After collecting the fishes we took photographs to record live coloration and kept them in crushed ice immediately to kill the fishes without unnecessary pain. After killing the fish pectoral and pelvic fins or muscle from the right caudal peduncle were removed from each specimen and fixed in 100% ethanol. The remainder of each specimen was fixed in 10% formalin for later identification. Five to 10 mg of each ethanol-fixed tissue sample was added to a 1.5 ml microfuge tube, and, following thorough homogenization with a sharp pair of scissors, genomic DNA was extracted using either the AquaPure Genomic DNA Isolation Kit (Bio-Rad, CA) or the DNeasy Blood & Tissue Kit (Qiagen, CA). Genomic DNA was extracted according to the manufacturer’s protocols. Identification of all specimens was verified by M. A. and S. M. The seven littoral species collected were *Lithochromis rubripinnis* Seehausen et al. 1998 [21], *L. rufus* Seehausen et al. 1998 [21], *Neochromis rufocaudalis* Seehausen et al. 1998 [21], *N. greenwoodi* Seehausen et al. 1998 [21], *N. omnicaeruleus* Seehausen et al. 1998 [21], *Haplochromis* (*Paralabidochromis*) *sauvagei* (Pfeffer, 1896) [22] (more specifically, this species was *H.* sp. “rockkribensis” sensu Seehausen, 1996 and; not *H. sauvagei* sensu Greenwood, 1957 [23] and Barel et al., 1977 [24]; see Seegers [25] for more information), and *Mbipia mbipi* Seehausen et al. 1998 [21]. In this study, we use conventional species names to simplify cross-referencing with other studies. Although *L. rufus* has been previously described as a rock-dwelling species, we caught individuals of this species in vegetation zones containing reed grass and/or papyrus. The other six species are territorial rock-dwelling species. Therefore, we consider *L. rufus* to occupy a different habitat from the other rock-dwelling species. Among our specimens, certain individuals belonging to *Lithochromis* or *Haplochromis* could not be identified at the species level, and we refer to these as *Lithochromis* sp. and *Haplochromis* sp., respectively. In addition, our samples included five specimens of *Pundamilia macrocephala*.

**Figure 1:**
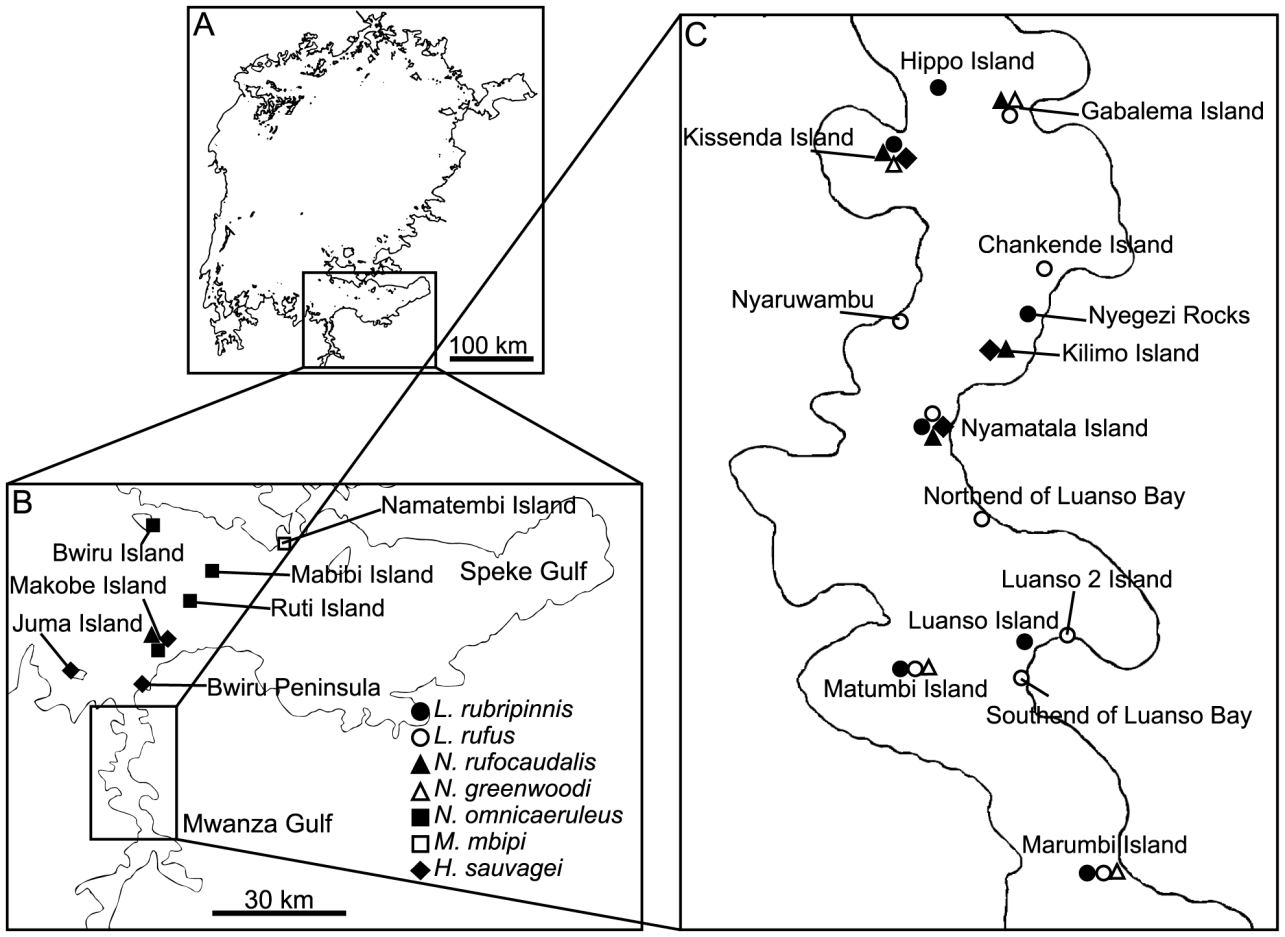
Sampling locations of the cichlids. Different species are represented by different symbols as indicated in Panel B. Panel A: Lake Victoria. Panel B: the sourhtern part of Lake Victoria. Panel C: Mwanza Gulf.

The total number of individuals sampled and the number of locations from which specimens were collected are summarized in Table 1. Sampling details are shown in Fig. 1 and Table S1. Specimens caught in the same location were considered to be part of the same population.

**Table 1:** Numbers of individuals typed and sequenced in each species.

	Habitat^a^	Microsatelliteloci	mtDNA control region
*H.* (*Y.*) *pyrrhocephalus*	P	289	166
*H.* (*Y.*) *laparogramma*	P	89	36
*L. rubripinnis*	R	61	66
*L. rufus*	V	112	128
*M. mbipi*	R	13	13
*N. rufocaudalis*	R	81	81
*N. greenwoodi*	R	77	77
*N. omnicaeruleus*	R	46	46
*H.* (*P*.) *sauvagei*	R	103	103
*Haplochromis* spp.	R	25	22
*Lithochromis* spp.	R	5	5
*P. macrocephala*	R	5	5
total		906	748

a P: pelagic, V: vegetation zone, R: rocky-shore.

### Amplification of Microsatellite Loci and Genotyping

We amplified 12 microsatellite loci from each sample using primers developed by Maeda et al. [20]. Forward primers used for the microsatellite markers were 5′-labeled with 6-FAM, NED, PET, or VIC dyes (Applied Biosystems, CA). Multiplex polymerase chain reactions (multiplex PCRs) were performed to amplify the target fragments using the QIAGEN Multiplex PCR Kit (Hilden, Germany). PCR amplifications were performed in a final reaction volume of 6.25 µL [3.125 µL 2× QIAGEN Multiplex PCR Master mix, 0.625 µL 10× Primer Mix (2 µM), 1.5 µL RNase free water and 1 µL diluted DNA (containing <10 ng of genomic DNA)]. The PCR amplification conditions were as follows: genomic DNA was denatured for 15 min at 95°C, followed by 35 cycles of denaturation for 30 s at 94°C, annealing for 1 min and 30 s at 55°C, and extension for 1 min at 72°C. Extension was completed using a final incubation for 30 min at 60°C. The PCR products were run on an ABI3100 automated sequencer (Applied Biosystems, CA) with a GeneScan™ –500 LIZ™ Size Standard (Applied Biosystems, CA) and genotyped using GeneMapper® Software Version 4.0 (Applied Biosystems, CA). To combine the results with those of the previous study [15], the same bin sets were used for both experiments.

### Mitochondrial DNA (mtDNA) Amplification and Sequencing

We amplified the mitochondrial control region using the primer pair SNmt-UP1 (5′-TAAAATCCTTCCTACTGCTTCA-3′) and SNmt-LP1 (5′-TCAAACAAAATATGAATAACAAACA-3′) as described by Nagl et al. [26] These primers are specific to the tRNA^Pro^ tRNA^Thr^ gene and the 3′-end of the control region, respectively. The amplification products encompassed nearly the entire control region (approximately 850 bp). PCR amplification was performed using either Ex*Taq*™ (TaKaRa, Ohtsu, Japan) or GoTaq® DNA polymerase (Promega, WI) according to the manufacturer’s recommendations. The PCR amplification conditions were as follows: DNA was denatured for 2 min at 94°C, followed by 30 cycles of denaturation for 40 s at 94°C, annealing for 30 s at 58°C, and extension for 1 min at 72°C. Extension was completed using a final incubation for 10 min at 72°C.

Two microliters of purified PCR product was used as a template in the cycle sequencing reactions. The primers used for sequencing were the two PCR primers SNmt-UP1 and SNmt-LP1 and two internal primers int-F (5′-CCTTTCATTTGACATCTCA-3′) and int-R2 (5′-CACACGCTGGAAAGAACGCC-3′). When DNA sequencing results were ambiguous, two additional internal primers, int-F2 (5′-CCACCATCCTATTTACATCCCT-3′) and int-R (5′-TCAACTGATGGTGGGCTCTT-3′), were used for further sequencing. The reaction mixture for the cycle sequencing consisted of 1.0 µL of each primer (1.6 µM), 1.25 µL Half BigDye (Genetix, New Milton, UK), 0.75 µL BigDye (Applied Biosystems, CA) and 5.0 µL of sterilized water. The annealing temperature for the cycle sequencing reactions was adjusted to 50°C. The DNA products were purified using ethanol/sodium-acetate precipitation, resuspended in 15 µL Hi-Di™ Formamide (Applied Biosystems, CA), and analyzed using an ABI PRISM 3100 capillary DNA sequencer (Applied Biosystems, CA). All sequences obtained in this study have been deposited within the DNA Data Bank of Japan (DDBJ) under the accession numbers [DDBJ: AB762784–AB 763333].

### Data Analyses

In the following analyses, we included data obtained by Maeda et al. [15] for two pelagic species – *Haplochromis* (*Yssichromis*) *pyrrhocephalus* and *H.* (*Y.*) *laparogramma* – with the data from the seven species described above. For the STRUCTURE and haplotype network analyses, we also included data from the *Lithochromis* spp. and *Haplochromis* spp. specimens that could not be identified at the species level. For the remaining analyses, classification at the species level was necessary, and therefore, data from these specimens were not included. Furthermore, because the sample size for *P. macrocephala* was small (five), these data were only used for the STRUCTURE and haplotype network analyses.

First, to determine the population structure of the whole sample set, we applied a Bayesian model-based clustering method to the microsatellite data, which was implemented in STRUCTURE Version 2.3.3 [27], [28]. Briefly, the program assumed a certain number of populations (*K*) and assigned each individual to one of the populations based on its multi-locus genotype. In our analyses, we applied the admixture model, which assumed that each individual might have mixed ancestry. We assumed *K* to be between 1 and 20, and we did not specify the origins of the samples. Each run consisted of 10,000 burn-in iterations, followed by 100,000 iterations to collect data. Other than these variables, the default program settings were used. We executed 20 runs for each *K* value, computed the averages of the estimated Log probabilities of the data (ln P[*D*]), and calculated Δ*K* for each *K* using the method of [29]. It has been suggested that Δ*K* can be used to detect the uppermost hierarchical level of genetic structure.

Sequences from the mitochondrial control region were edited and aligned by eye using the computer program Se-Al [30]. To this alignment, we added sequence data from 51 haplotypes of Lake Victoria, Lake Kivu and Lake Victoria obtained by Nagl et al. [26] and Verheyen et al. [4]. Gaps were included in the sequences as information, as indels reflect the evolutionary history of the species. We constructed a haplotype network of these sequences using the program TCS [31]. Alternative branching orders in the TCS-generated network were assessed using the maximum parsimony method and the software program PAUP* 4.0b10 [32]. Only connections between haplotypes favored by the maximum parsimony criterion were used.

Next, to determine whether there were species-level differentiations of the mitochondrial and microsatellite loci within each group (defined in the Results section), we carried out Analysis of Molecular Variance (AMOVA) [33] on the eight species for which multiple populations were sampled. The hierarchy of the analysis was species/populations for the mtDNA and species/populations/individuals for the microsatellite markers. We used the software programs GenAlEx Version 6.4 [34] for the mitochondrial data and Arlequin Version 3.5 [35] for the microsatellite data. Between populations, we also estimated *R*_ST_ [36] for the microsatellite data and *F*_ST_ [37] for the mitochondrial data using Arlequin Version 3.5 and DNAsp 5.0 [38], respectively. Under the assumption of the symmetric stepwise mutation model for the microsatellite loci, *R*_ST_ measures the same quantity as *F*_ST_ defined by [37] for nucleotide sequences [36] does. To evaluate the significance of differentiation, we used permutation tests for *R*_ST_ for the microsatellite data and *S*_nn_ [39] for the mitochondrial data. Significance levels for multiple testing were corrected using the Bonferroni procedure. Furthermore, we conducted the Mantel test of association [40] using GeneAlex to examine the relationship between linearized *F*_ST_ (or *R*_ST_), *F*_ST_/(1−*F*_ST_) [41], and geographic distance. The geographic distance was measured as the shortest waterway distance between location pairs.

The basic genetic parameters of variation within species were calculated. For the mitochondrial data, we estimated nucleotide diversity π [42], Watterson’s estimator of the population mutation rate θ_W_ [43], and Tajima’s *D* [44] using DNAsp 5.0 [38]. We also estimated the parameters of the demographic expansion model of Schneider et al. [45] using their method, which was implemented in Arlequin. In this method, population size is assumed to increase quickly from *N*_0_ to *N*_1_ *t* generations ago, with the estimated parameters θ_0_ = 2*N*_0_ *u*, θ_1_ = 2*N*_1_ *u* and *T*_0_  = 2*ut* (where *u* is the mutation rate). Goodness of fit for the model was evaluated using the estimated parameters. For the microsatellite data, we computed the expected heterozygosity (*H*_E_) and Wright’s inbreeding coefficient (*F*_IS_) using Arlequin.

## Results

In total, data for the 12 microsatellites and the mitochondrial control region from 906 and 748 individuals of cichlids, respectively, were used for the analysis.

### Population Structure Inferred by Structure

**Figure 2:**
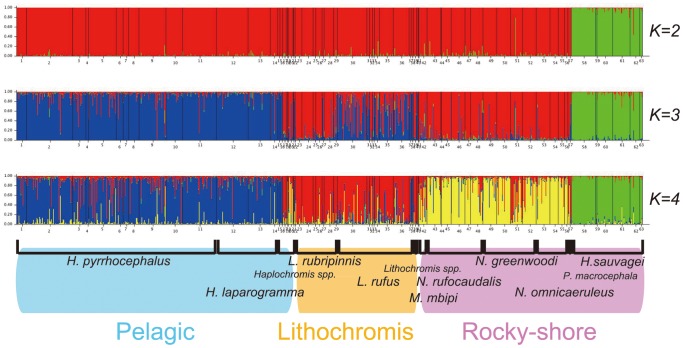
Results of STRUCTURE analyses of the entire sample set with *K* = 2–4. The grouping of the species is shown at the bottom. Speccies delimitation is indicated by the vertical bars above the species names.

To infer the population structure of the whole sample set, including the two pelagic species, we ran the STRUCTURE program [27] using data from the 12 microsatellite loci, assuming the number of populations (*K*) to range from 1 to 20. The estimated log probability of the data (ln P[*D*]) increased as *K* was incrementally raised from 1 to 4, stayed approximately constant until *K* reached 11, and then decreased rapidly as *K* was increased further (data not shown). The maximal ln P[*D*] value was reached when *K* = 8. The modal value for the index as defined by Evanno et al. [29], Δ*K*, was reached when *K* = 2. Therefore, the number of populations at the uppermost hierarchical level of population structure appears to equal two [29]. However, further subdivisions were apparent as the number of populations was increased. Individual assignments are shown in Fig. 2 for *K* = 2, 3, and 4. With a few exceptions, individuals belonging to the same species were classified into the same emerging groups as the *K* value was increased. When *K* = 2, the rock-dwelling species *Haplochromis* (*Paralabidochromis*) *sauvagei* separated from the other eight species. When *K* = 3, the two pelagic species, *H.* (*Yssichromis*) *pyrrhocephalus* and *H.* (*Y.*) *laparogramma*, separated from the remaining six littoral species, although part of their genetic components were shared by some *Lithochromis*. When *K* = 4, the six littoral species separated into two groups, one group consisting of the species belonging to the genus *Lithochromis*, *L. rubripinnis, L. rufus* and *L.* spp., and the other group consisting of four rock-dwelling species, *Neochromis rufocaudalis, N. greenwoodi, N. omnicaeruleus* and *Mbipia mbipi*. Because part of the species boundary became obscure when *K* ≥5 (data not shown), we did not consider these cases any further. Thus, we restrict our attention to the four groups identified when *K* = 4 and refer to them as the following: (1) pelagic [*H.* (*Y.*) *pyrrhocephalus and H.* (*Y.*) *laparogramma*], (2) *Lithochromis* (*L. rubripinnis and L. rufus*), (3) rocky-shore 1 (*H.* (*P.*) *sauvagei*), and (4) rocky-shore 2 (*N. rufocaudalis, N. greenwoodi, N. omnicaeruleus* and *M. mbipi*). Although our specimens of *L. rufus* were mainly caught in vegetation zones, specimens of *L*. *rubripinnis* were found along rocky shores. Therefore, these two species could not be assigned to a single habitat. Thus, the nine studied species were genetically classified into four groups, three of which only contained species from a single habitat. Note that a small number of individuals classified as *Lithochromis* spp. were also grouped genetically into the *Lithochromis* group, although those classified as *Haplochromis* spp. could not be unambiguously assigned to any one group.

### Haplotype Network of the Mitochondrial Control Region

**Figure 3:**
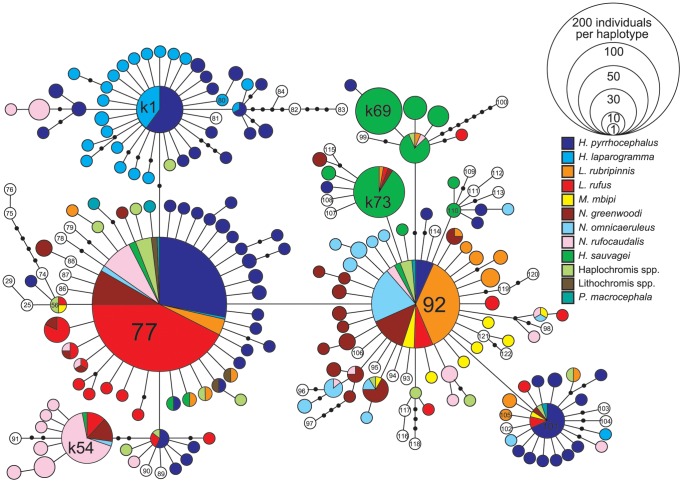
Haplotype network of the mitochondrial control region. Different species are represented by different colors. *H. (Y.) pyrrhocephalus* and *H. (Y.) laparogramma* are pelagic. *L. rufus* lives in the vegetation zone and the remainig species live in rocky shores. The size of the the circle shows the number of the samples having the haplotype.

The haplotype network was reconstructed using sequence data from the mitochondrial control region (Fig. 3). Ten species, including *Pundamilia macrocephala*, the *Lithochromis* spp. and the *Haplochromis* spp., are represented by 12 different colors in Fig. 3. We employed the haplotype designations defined by Verheyen et al. [4] for the previously characterized haplotypes. Many new haplotypes were identified in the present study, which have been numbered from k1 to k175.

The sequences from these nine species were not monophyletic, as has been previously noted for the cichlids of Lake Victoria by Verheyen et al. [4]. However, characteristic distributions of haplotypes can be observed for some of the species. For example, although the haplotypes of the two pelagic species (blue and light blue) were scattered throughout the haplotype network, the majority of them were concentrated around haplotype 77 and haplotype k1. On the other hand, haplotypes of the two *Lithochromis* species (red and orange) were mostly clustered around haplotypes 77, 92 and other closely related haplotypes. Another striking case was that of *H.* (*P.*) *sauvagei* (*H.* sp. “rockkribensis”), whose haplotypes (green) were generally located near haplotype 92 and were mostly species specific. On the other hand, the haplotypes of another rock-dwelling species, *N. rufocaudalis* (pink), were widely distributed throughout the network. Therefore, species differed in the distributions of their mitochondrial haplotypes throughout the haplotype network, and the haplotypes of *H.* (*Y.*) *laparogramma*, *H.* (*P*.) *sauvagei, L. rubripinnis* and *L. rufus* clustered in a similar manner to clusters observed with the STRUCTURE-based groupings based on the nuclear microsatellite markers.

### AMOVA and Analyses based on *F*_ST_ and *R*_ST_

To evaluate genetic differentiation between species within groups, we carried out AMOVA within each group and estimated the variation both within and between species using the mitochondrial and microsatellite data. These analyses were carried out for the pelagic, *Lithochromis* and rocky-shore 2 groups, which contained multiple species, each of which was sampled at multiple locations. These results are shown in Table 2. For all groups, the variation between species was significant. The relative variance components were 19.31∼27.87% for mtDNA and 1.14∼4.81% for microsatellites (all *P*<0.05). Therefore, species appeared to be genetically divergent within each group. Indeed, pairwise *F*_ST_ values for the mitochondrial locus and *R*_ST_ values for the microsatellite loci between species were mostly significant, as shown in Table S2. Furthermore, the variation between populations within species was significant for all groups. Relative variance components were 5.56∼31.52% for mtDNA and 0.33∼3.32% for microsatellites (all *P*<0.01).

**Table 2:** Results of AMOVA at mitochondrial and nuclear loci.

	pelagic			*Lithochromis*			rocky-shore 2		
	df	% variation	P value	df	% variation	P value	df	% variation	P value
**mtDNA**									
between species	1	22.51	0.021	1	27.87	0.000	3	19.31	0.006
between populations	12	21.57	0.000	17	5.56	0.000	16	31.52	0.000
within populations	188	55.92		175	66.58		287	49.17	
**microsatellite**									
between species	1	1.14	0.005	1	1.15	0.001	3	4.81	0.000
between populations	12	0.33	0.001	14	1.08	0.000	15	3.32	0.000
between individuals	364	2.35	0.000	155	1.79	0.011	287	1.45	0.016
Within individuals	378	96.18		171	95.99		306	90.42	

**Figure 4:**
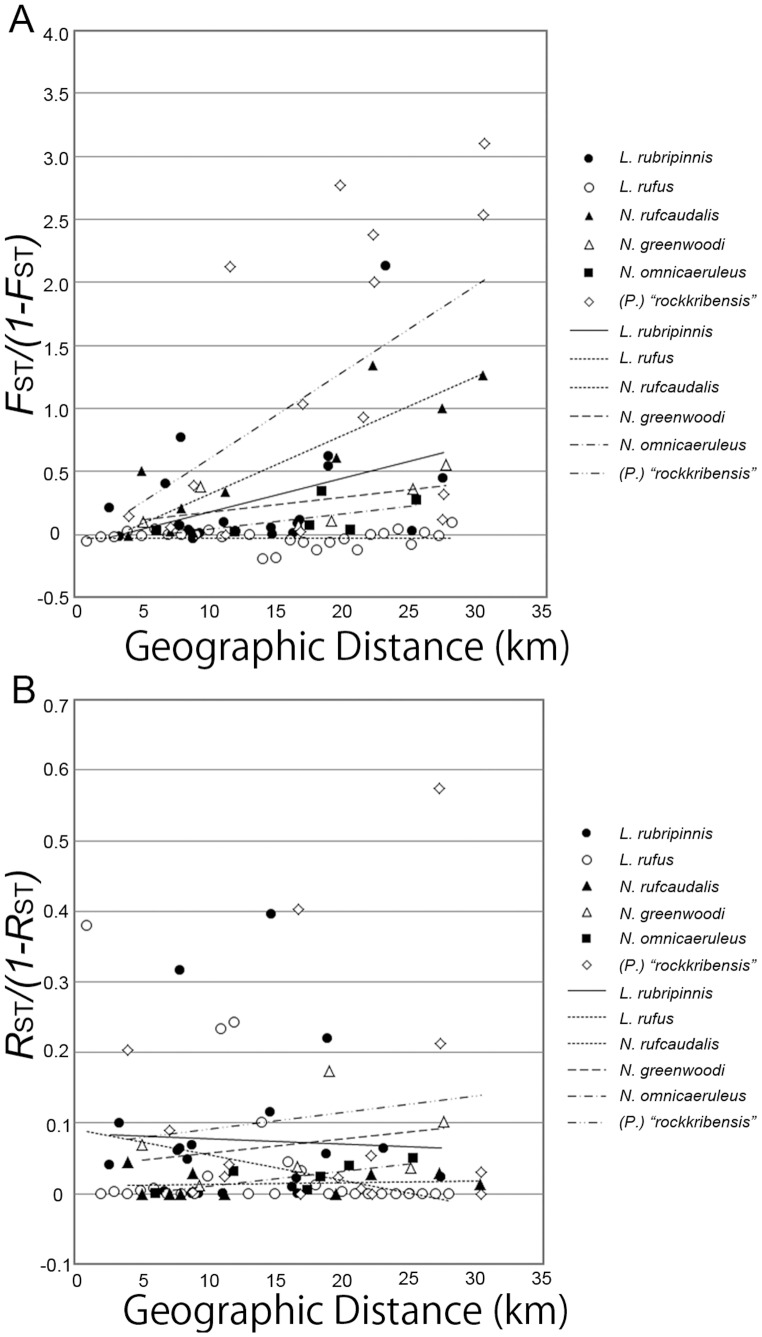
The relationships between geographical distance and genetic differentiation. Pane A: mitochondrial. Panel B: nuclear microsatellite loci.

We also estimated pairwise *F*_ST_ values for the mitochondrial locus and *R*_ST_ values for the microsatellite loci between populations within species, and these results are shown in Table S3. At the microsatellite loci, significant differentiation was found between one or more pairs of populations in only two species, *N. greenwoodi* and *L. rubripinnis*, and, in general, estimates of *R*_ST_ were lower than those for *F*_ST_ at the mitochondrial locus. For example, in *N. greenwoodi*, the Gabalema population was significantly differentiated, albeit weakly, from the other populations with respect to the microsatellite loci (*R*_ST_ = 0.065–0.148, *P* = 0.001–0.003). On the other hand, for the mitochondrial control region, we found significant differentiation between populations in all species, with the exception of *H. pyrrhocephalus*, *H. laparogramma* and *L. rufus*, and estimates of *F*_ST_ were generally high. For example, in *H.* (*P.*) *sauvagei*, seven of the 15 population pairs showed significant differentiation (*F*_ST_ = 0.278–0.756, *P* = 0.0000). For the *Lithochromis* and rocky-shore 2 species in which four or more populations were sampled, we plotted the linearized *F*_ST_ (or *R*_ST_), *F*_ST_/(1–*F*_ST_), (shown in Fig. 4) and tested isolation by distance using the Mantel test. Isolation by distance was found for *N. rufocaudalis* (*P* = 0.043) and *H.* (*P.*) *sauvagei* (*P* = 0.051) using the mitochondrial control region data, though it was not significant in the latter species. Weak isolation by distance was also observed for the mitochondrial region in *L. rubripinnis*, although this was not significant (*P* = 0.066).

### Diversity Statistics within Species and Inferences on Expansion

We estimated various diversity statistics for each species, which are shown in Table 3. *N. rufocaudalis* had the highest nucleotide diversity in the mitochondrial control region (π = 0.00481), whereas *L. rufus* had the lowest diversity (π = 0.00118), although both species had high levels of diversity at the microsatellite loci. *H.* (*Y.*) *pyrrhocephalus* had the highest θ_W_ value (0.01142), whereas *H.* (*P.*) *sauvagei* had the lowest θ_W_ value (0.00328). *F*_IS_ was not significantly different from zero in any species after Bonferroni correction (data not shown). In all species, Tajima’s *D* values were negative, and these results were significant for the two pelagic species, two *Lithochromis* species, *M. mbipi*, and *N. omnicaeruleus*.

**Table 3:** Statistics of population diversity and estimates for population size change for the 9 species.

	Hp^a^	Hl^a^	Lrub^a^	Lruf^a^	Mm^a^	Nr^a^	Ng^a^	No^a^	Hs^a^
**mtDNA**									
*n*	166	36	66	128	13	81	77	46	103
*S*	57	29	18	24	10	28	23	23	15
*π*	0.00266	0.00226	0.00145	0.00118	0.00221	0.00481	0.00271	0.00263	0.00236
θ_W_	0.01165	0.00823	0.00430	0.00504	0.00367	0.00642	0.00533	0.00595	0.00328
Tajima’s *D*	–2.412**	–2.504***	–2.071*	–2.317**	–1.834*	–0.862ns	–1.640ns	–1.903*	–0.905ns
θ_0_ b	0.353	0.005	0.030	0.000	0.000	0.005	0.012	0.000	0.000
θ_1_ b	17.412	∞	7.437	10.833	∞	7.668	∞	∞	6.455
*T*_0_ ( = 2*ut*)	3.342	2.199	1.535	0.719	2.295	6.207	2.277	2.250	3.840
time (years)^c^	82279	54139	37791	17702	56502	152814	56059	55394	94539
goodness of fit^d^	0.889	0.633	0.997	0.137	0.453	00.489	0.014	0.546	0.067
**microsatellite**									
*n*	289	89	61	112	13	81	77	46	103
heterozygosity	0.750	0.745	0.731	0.779	0.744	0.741	0.729	0.768	0.697

a Hp, *H*. (*Y*.) *pyrrhocephalus*: Hl, *H*. (*Y*.) *laparogramma*: Lrub, *L*. *rubripinnis*: Lruf, *L*. *rufus*: Mm, *M. mbipi*: Nr, *N. rufocaudalis*: Ng, *N. greenwoodi*: No, *N. omnicaeruleus*: Hs, *H.* (*P.*) *sauvagei*

b Parameters of the model by Schneider and Excoffier (1999).

c *u* = 2.3×10–8 per year per base pair was assumed.

d Results of goodness of fit for the predicted expansion model.

* significant at 5%,

** significant at 1%.

*** significant at 0.1%.

As negative values for Tajima’s *D* indicate recent demographic expansion, we used the method described by Schneider et al. [45] and implemented in Arlequin Version 3.5 [35] to estimate θ_0_ = 2*N*_0_ *u*, θ_1_ = 2*N*_1_ *u* and *T*_0_ = 2*ut* using the mitochondrial data. The results are shown in Table 3. Assuming an evolutionary rate of 2.3×10^−8^ per year per base pair in this region, as was employed by Samonte et al. [14], we estimated the absolute year of the start of expansion. For all species, current population size was estimated to be at least 30 times greater than the size of the population before expansion, and the start of expansion was estimated to have occurred between 17,000 and 95,000 years ago. Except for *N. greenwoodi* and *H. sauvagei*, fits of the expansion model were generally good.

## Discussion

### Hierarchic Genetic Grouping

In the present paper, we genetically examined nine cichlid species from Lake Victoria using mitochondrial and microsatellite markers to determine the genetic structure of cichlid populations during early adaptive speciation. More specifically, we asked whether a hierarchical genetic structure exists within cichlid fish populations in Lake Victoria, and if so, how is this structure related to habitat and species?

Our analyses of microsatellite loci using STRUCTURE showed that the nine studied species could be genetically classified into four groups (Fig. 2): pelagic (*Haplochromis* (*Y.*) *pyrrhocephalus* and *H.* (*Y.*) *laparogramma*), *Lithochromis* (*L. rubripinnis* and *L. rufus*), rocky-shore 1 (*H.* (*P.*) *sauvagei*), and rocky-shore 2 (*Neochromis rufocaudalis*, *N. greenwoodi, N. omnicaeruleus* and *Mbipia mbipi*). Note that the rocky-shore species *L. rubripinnis* is included in the *Lithochromis* group but is not included in the rocky-shore group. In addition, two rocky-shore groups, the first consisting of *H.* (*P.*) *sauvagei* and the second consisting of the four remaining rocky-shore species, were differentiated genetically. Therefore, with the caveat that our samples were limited to only nine of the approximately 700 species found within the lake, we conclude that a hierarchical genetic structure of species groups, species and populations exists in cichlid fish from Lake Victoria and that this structure is partially correlated with their respective habitats. Some of the groupings (e.g., pelagic) are consistent with the scenario proposed by Danley et al. [9], which posits that adaptive radiation of cichlid fish in Lake Malawi occurred first by adaptation to different habitats. Alternatively, this pattern can be explained by the higher level of gene flow between species that diverged earlier but still live together in the same habitat [46]. In this case, speciation may not have occurred first by adaptation to different habitats. To discriminate between recent separation of populations and high levels of migration between them as a cause of the genetic similarity of the species in the same habitat, more detailed analyses such as those by IMa [47] using multi-locus sequence data would be necessary.

Another notable feature of this grouping is that, with the exception of *Haplochromis*, individuals of a given genus were confined to individual groups. This finding is consistent with the genus-level clustering of cichlids in Lake Victoria as shown by Bezault et al. [17] using AFLP markers. Additionally, with the exception of a small number of individuals that includes those identified as either of the *Haplochromis* species, individuals belonging to the same species as judged from their morphology were classified into the same group as defined by the 12 microsatellite markers.

The differentiation of the rocky-shore 1 group from the other cichlids within Lake Victoria has been known for some time. This group consists of *H.* (*P.*) *sauvagei*, which was previously known as *H. sp.* “rockkribensis” (the species previously called *H. sauvagei* is now known as *H. fischeri* Seegers, 2008). Nagl et al. [26] found that the mitochondrial haplotypes of this species belonged to subgroup VD, whereas the haplotypes of all the other cichlids in Lake Victoria belonged to subgroup VC. Moreover, Samonte et al. [14] estimated that this species diverged from the other cichlids in Lake Victoria approximately 41,300 years ago, whereas the other cichlids in the lake diverged from each other approximately 13,800 years ago, a period during which desiccation of the lake is thought to have occurred [48]. This result may indicate that the rocky-shore 1 group (*H.* (*P.*) *sauvagei*) has a unique evolutionary origin and that adaptation to the rocky-shore habitat during the early stage of the adaptive radiation in Lake Victoria might be represented by the rocky-shore 2 group.

We could also detect significant genetic differentiation between species within each group using AMOVA of the mitochondrial and microsatellite data, although the levels of differentiation differed between the markers and groups (Table 2). Nonetheless, the correct assignment of individuals to species groups using STRUCTURE analysis of the microsatellite data was not possible (data not shown). Although the locations of our sample collections were restricted to the Mwanza Gulf and the surrounding areas, sampling points for each species were scattered throughout the region, and different species from the same groups were occasionally sampled at the same location, as shown in Fig. 1. Therefore, differentiation between species beyond the differentiation observed between populations within species was observed at the neutral marker loci, although the levels of differentiation were generally very low at the microsatellite loci.

Our finding that significant genetic differentiation exists between species beyond what was observed between populations within species does not agree with the results of Samonte et al. [14], who found that, with the exception of *H.* (*P.*) *sauvagei*, interpopulation genetic distances within species were similar to those observed between species in Lake Victoria. Additionally, Konijnendijk et al. [46] have shown that allopatric conspecific populations were more strongly differentiated than sympatric heterospecific populations of closely related species. Finally, Elmer et al. [13] stated that current markers and methods were not sufficient to differentiate between biological species within Lake Victoria. Our contradictory findings might be explained by differences inherent to the species used in this study, differences in the marker type (e.g., microsatellites versus nuclear gene sequences in the case of Samonte et al. [14]), numbers of markers (see [17], [18]) or the differences in the geographic distances between surveyed populations. Indeed, our population samples were generally separated by 30 km or less, whereas the populations used by Samonte et al. [14] were separated by up to 350 km. Therefore, differentiation between populations may be underestimated in our study, as our samplings did not cover the entire range of each species. However, as the populations of each species were scattered throughout the studied region and were not concentrated geographically (Fig. 1), we think that differentiation in neutral marker loci between species beyond what is observed in populations is a real phenomenon in the species studied here. This finding is in agreement with a recent study by Wagner et al. [18], who used RAD markers to show reciprocal monophyly of the species in Lake Victoria.

### Population Expansion

Estimates of Tajima’s *D* values for the mitochondrial control region were negative for all species and highly significant in species belonging to the pelagic and *Lithochromis* groups (Table 3). The negative values of Tajima’s *D* were caused by many low-frequency haplotypes that differed by one base pair from the major haplotypes (77, 92, 101 and k1 in Fig. 3). Because these results indicated recent population expansions, we estimated the time of the expansions using the method described by Schneider et al. [45]. If we assume the mutation rate per base pair per year in the mitochondrial control region to be 2.3×10^−8^, as used by Samonte et al. [14] for the cichlids of Lake Victoria, the expansion time was estimated to be between 17,000 and 83,000 years ago for the species in the pelagic and *Lithochromis* groups, though we need to note that confidence intervals for the estimates from single locus data are usually very large. Also because the method [45] assumes a random mating population, which was violated in some of the studied species as shown in Fig. 4, some of the estimates might not be reliable.

Based on the microsatellite data, Elmer et al. [13] inferred that cichlid populations in Lake Victoria began to decline approximately 18,000 years ago, and they suggested that this decline corresponded to the desiccation of Lake Victoria hypothesized by Johnson et al. [48]. This decline may in fact correspond with the beginning of the expansion we estimated using mitochondrial markers [15], [16]. First, our estimate of an expansion occurring between 17,000 and 83,000 years ago might be an overestimate due to an acceleration of evolutionary rates during more recent periods, possibly due to inclusion of deleterious mutations, as has been previously suggested by Ho et al. [49] and Genner et al. [11]. Therefore, the beginning of the expansion could be closer to the estimate of 18,000 years ago proposed by Elmer et al. [13]. Second, a bottleneck event produces different patterns in neutrality-test statistics for mitochondrial genes compared with nuclear genes [50]. More specifically, values of Tajima’s *D* for mitochondrial genes quickly become negative following a bottleneck event, whereas values for nuclear genes stay positive for some time. Therefore, for a short period after the bottleneck event, mitochondrial genes may indicate a population expansion, whereas nuclear genes may indicate a population decline. Therefore, our assessment of population expansion may indeed be consistent with the findings of Elmer et al. [13].

### Population Structure within Species

At the mitochondrial control region, we observed significant differentiation between the populations of most species, although the levels of differentiation differed between species. Strong differentiation was observed in *H.* (*P.*) *sauvagei* and *N. rufocaudalis*, showing isolation by distance, whereas differentiation in *L. rufus* and *N. omnicaeruleus* was weak (Fig. 4). Furthermore, the levels of differentiation differed even among species within the same group (e.g., *N. rufocaudalis* and *N. omnicaeruleus*). In contrast, although differentiation between populations was significant at the microsatellite loci in the AMOVA analysis, most of the pairwise *R*_ST_ values between populations were not significant. This indicated that the levels of differentiation at those loci were very low and could be detected only when a large number of samples were analyzed together.

In Lake Malawi, although three pelagic species show little differentiation between populations separated by more than 100 km [51], rock-dwelling mbuna and non-mbuna species show much stronger differentiation [52]. On the other hand, some species inhabiting rocky shores in Lake Victoria showed very weak differentiation between populations (*L. rubripinnis* and *N. omnicaeruleus*) in our study. Other authors have compiled similar results [8], [53], [54]), although we do note that our samples were collected from an area approximately 30 km in diameter. The low levels of differentiation observed between populations of species living along rocky shores may indicate high mobility for those species. As species evolve lower mobility within this habitat, species may accumulate higher levels of differentiation between populations. On the other hand, these populations may show low differentiation due to the relatively recent dispersal of the species. Either way, the weak differentiation between populations of certain rocky-shore species stands in contrast to the strong differentiation found in rocky-shore species in other lakes [52] and may indicate a recent diversification of cichlids in Lake Victoria [2], [4].

## Conclusions

Based on the observation of low levels of differentiation and an overlap between mitochondrial and nuclear haplotypes [12], [26], the cichlid species of Lake Victoria have often been treated as genetically homogenous (e.g., [13]). However, as shown here, a clear hierarchical genetic structure can be seen in the cichlid fishes of Lake Victoria. Interestingly, the groupings were mostly consistent with the genus-level clustering, and some of the groups corresponded to different habitats. The habitat clustering found in some groups may be explained by the scenario proposed by Danley et al. [9] for Lake Malawi in which species first diverge based on habitat. However, recent gene flow between species in the same habitat can also explain the hierarchical structure. In addition, most species appear to be differentiated within each group, as has been recently shown by Bezault et al. [17] using AFLP markers and by Wagner et al. [18] and Keller et al. [19] using RAD markers. Finally, each species showed its own characteristic genetic structure, with either high or low levels of population differentiation. As this radiation process occurred recently, we were able to study this process more accurately than is possible with older radiations. Therefore, the cichlid fish of Lake Victoria provide a good opportunity to study adaptive radiation. Future studies that use larger numbers of nuclear markers will help us understand this process in greater detail.

## Supporting Information

Table S1 **Locations and numbers of samples for each species.**
(XLS)

Table S2 ***F*** **_ST_ estimated by mtDNA (bellow diagonal) and** ***R*** **_ST_ estimated by microsatellites (above diagonal0 between species.**
(XLS)

Table S3 ***F*** **_ST_ estimated by mtDNA (bellow diagonal) and** ***R*** **_ST_ estimated by microsatellites (above diagonal0 between populations within species.**
(XLS)
